# In Vitro Effects of Low Doses of β-Caryophyllene, Ascorbic Acid and d-Glucosamine on Human Chondrocyte Viability and Inflammation

**DOI:** 10.3390/ph14030286

**Published:** 2021-03-23

**Authors:** Elena Mattiuzzo, Alessia Faggian, Rina Venerando, Andrea Benetti, Elisa Belluzzi, Giovanni Abatangelo, Pietro Ruggieri, Paola Brun

**Affiliations:** 1 Department of Molecular Medicine, Histology Unit, University of Padova, 35121 Padova, Italy; elena.mattiuzzo@unipd.it (E.M.); alessia.faggian@unipd.it (A.F.); andrea.benetti.11@phd.unipd.it (A.B.); 2 Department of Molecular Medicine, University of Padova, 35121 Padova, Italy; rina.venerando@unipd.it; 3 Musculoskeletal Pathology and Oncology Laboratory, Department of Surgery, Oncology and Gastroenterology, University of Padova, 35128 Padova, Italy; elisa.belluzzi@unipd.it; 4 Orthopaedic and Traumatology Clinic, Department of Surgery, Oncology and Gastroenterology, University of Padova, 35128 Padova, Italy; pietro.ruggieri@unipd.it; 5 Faculty of Medicine, University of Padova, 35100 Padova, Italy; g.abatangelo@unipd.it

**Keywords:** β-caryophyllene, osteoarthritis, ascorbic acid, d-glucosamine, reactive oxygen species, inflammation, chondrocyte

## Abstract

β-caryophyllene (BCP), a plant-derived sesquiterpene, has been reported to have anti-inflammatory and antioxidant effects. The purpose of this study is to evaluate the effects of BCP in combination with ascorbic acid (AA) and d-glucosamine (GlcN) against macrophage-mediated inflammation on in vitro primary human chondrocytes. Changes in cell viability, intracellular ROS generation, gene expression of pro-inflammatory mediators, metalloproteinases (MMPs), collagen type II and aggrecan were analyzed in primary human chondrocytes exposed to the conditioned medium (CM) of activated U937 monocytes and subsequently treated with BCP alone or in combination with AA and GlcN. The CM-induced chondrocyte cytotoxicity was reduced by the presence of low doses of BCP alone or in combination with AA and GlcN. The exposure of cells to CM significantly increased *IL-1β, NF-*κ*B*1 and *MMP-13* expression, but when BCP was added to the inflamed cells, alone or in combination with AA and GlcN, gene transcription for all these molecules was restored to near baseline values. Moreover, chondrocytes increased the expression of *collagen type II* and *aggrecan* when stimulated with AA and GlcN alone or in combination with BCP. This study showed the synergistic anti-inflammatory and antioxidative effects of BCP, AA and GlcN at low doses on human chondrocyte cultures treated with the CM of activated U937 cells. Moreover, the combination of the three molecules was able to promote the expression of *collagen type II* and *aggrecan*. All together, these data could suggest that BCP, AA and GlcN exert a chondro-protective action.

## 1. Introduction

Osteoarthritis (OA) is a progressive inflammatory degenerative whole-joint disease with an increasing prevalence due to aging of the population [1,2]. Macrophage-mediated inflammation is considered one of the main drivers of both OA development and progression, and causes the release of proinflammatory cytokines, such as interleukin-1β 1 (IL-1β), and reactive oxygen species (ROS) [3,4]. These molecules induce chondrocytes to synthesize other inflammatory mediators that upregulate cytokines and increase matrix metalloproteinase (MMP) activity leading to cartilage disruption, mainly due to the imbalance between synthesis and degradation of extracellular matrix (ECM) molecules, in favor of degradation [1,2,3,4]. The main components of ECM are aggrecan and type II collagen, which in OA are degraded by MMP-13. Moreover, it has been shown that OA chondrocytes display epigenomic alterations leading to an alteration of nuclear factor κB (NF-κB) activation [5]. Activated transcriptional factor NF-kappa B (NF-κB) translocates into the nucleus where it binds to DNA, activating transcription of genes encoding proteins involved in inflammation responses. Therefore, it has been shown to play a key role in the acquisition of inflammatory phenotype and cytokine-induced secretion of MMPs by chondrocytes [6,7].

Current OA therapies include the use of corticosteroids (CSs) and nonsteroidal drugs, which have well-demonstrated anti-inflammatory effects [8,9]. However, adverse effects of these pharmacological treatments on cell viability and cartilage loss remain a concern. The lack of satisfactory effects of traditional drug therapy in controlling OA pain and progression could explain the increasing use of self-treatments such as d-glucosamine (D-GlcN) and other “nutraceuticals”. In particular, chondroitin sulphate and GlcN are suggested for the maintenance of normal joint cartilage in patients affected by OA [10].

It is therefore necessary to study new treatments and explore new molecules to be used alone or in combination, capable of modulating the production of oxidative and proinflammatory molecules, whilst also exerting a chondro-protective action on articular tissue [11,12]. One such molecule, the plant-derived bicyclic sesquiterpene β-caryophyllene (BCP), as recently reported, binds to CB2 (one of the two cannabinoid receptors, CB1 and CB2), and among its wide range of biological activities, exerts an anti-inflammatory effect [13], decreasing the expression of MMPs and IL-1β in human chondrocyte cultures [14]. In fact, cannabinoid receptors are constitutively expressed not only by immuno-derived cells, but also by neural cells, chondrocytes, osteoblasts and adipocytes [15,16,17,18]. Therefore, the use of CB2 receptor agonists represents an attractive treatment of OA as anti-inflammatory and chondro-protective drugs. Indeed, in a recent study it was demonstrated that BCP significantly ameliorates the severity of arthritis in mice, reducing the expression of proinflammatory cytokines through a cross-talk between CB2 and the peroxisome proliferator-activated receptor (PPAR)-γ receptor [14]. The anti-inflammatory effect of cannabinoids may be mediated by the nuclear hormone family PPAR, either directly or through CB2 receptors [16,19,20]. On these bases, we hypothesize that BCP’s pharmacological effects could be improved by combining this molecule with other antioxidants, such as ascorbic acid (AA) and/or chondro-protective molecules, such as GlcN. GlcN is considered to be a regulator of bone and cartilage metabolism and a nutraceutical that has been proven to be effective in OA patients [10]. In fact, GlcN is a substrate for the synthesis of glycosaminoglycans (GAGs), improving the production of cartilage proteoglycans such as aggrecan. It was also demonstrated that GlcN exerts anti-inflammatory and anticatabolic effects at high concentrations, inhibiting the expression of MMPs, aggrecanases and NF-kB [21,22]. Moreover, AA is able to decrease the oxidative stress related to OA progression and increase the synthesis of proteoglycans by chondrocytes [23,24].

To gain new insights into the activity of BCP, its antioxidant, anti-inflammatory and chondro-protective effects, the present study aims to explore its effects on viability and proinflammatory molecule expression in the presence or absence of AA and GlcN on human chondrocytes in an in vitro inflammatory model of phorbol 12-myristate 13-acetate (PMA)–lipopolysaccharides (LPS)-stimulated U937 human monocytes.

## 2. Results

### 2.1. The Evaluation of the Effects of BCP, Alone or in Combination with AA and GlcN on CM-Induced Chondrocyte Toxicity

**Figure 1:**
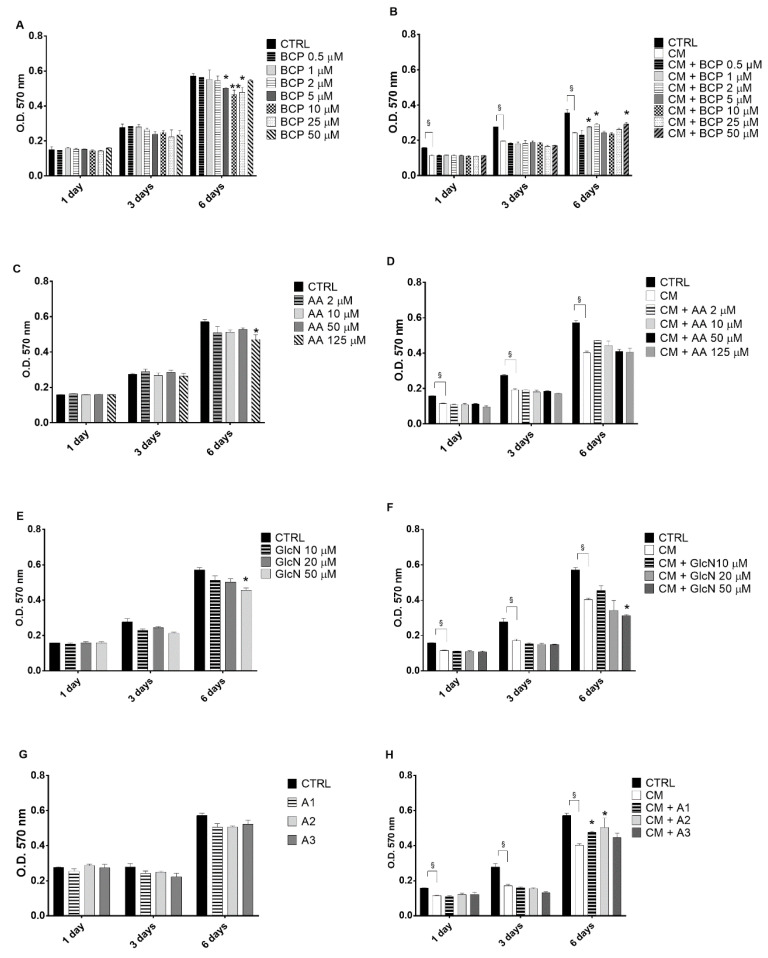
Time-dependent effects of BCP, AA and GlcN, alone or in combination, on human chondrocyte viability. Cells (7000/cm^2^) were seeded in 96-well culture dishes exposed or not for 24 h to the conditioned medium (CM) of activated U937 and then treated at the indicated concentrations. (**A**) BCP; (**B**) BCP after CM exposure; (**C**) AA; (**D**) AA after CM exposure; (**E**) GlcN; (**F**) GlcN after CM exposure; (**G**) AA, GlcN and BCP mixture (A1, A2, A3); (**H**) AA, GlcN and BCP mixture (A1, A2, A3) after CM exposure. A1, 1 µM BCP, 2 µM AA and 10 µM GlcN; A2, 1 µM BCP, 10 µM AA and 20 µM GlcN; A3, 1 µM BCP, 50 µM AA and 20 µM GlcN. The MTT test was performed 1, 3 and 6 days after treatment. Data are reported as mean ± SE of three independent experiments. Statistical differences based on ANOVA test with multiple comparisons. CTRL: untreated cells; CM: cell treated with CM of activated U937 cells. * *p* < 0.05 and ** *p* ≤ 0.01 vs. CM-treated cells. § *p* < 0.05, CM-treated cells vs. untreated cells.

In Figure 1, typical results of primary human chondrocytes cultivated in the presence of different concentrations of BCP, AA and GlcN, alone or in combination, were reported. BCP, at all the tested concentrations, did not affect cell proliferation after 1 and 3 days of treatment (Figure 1A). However, a significant reduction of viability was found in the presence of 5, 10 and 25 µM BCP after 6 days.

Pretreatment of the cells with the CM of activated U937 cells, which was used to induce an inflammatory response, significantly reduced the chondrocyte viability at all the tested times (1, 3 and 6 days) (Figure 1B). The treatment with low (1 and 2 µM) and high (50 µM) concentrations of BCP exerted a significant protective effect against CM-induced toxicity (*p* < 0.05) after 6 days. Intermediate BCP concentration did not exert any protective effects. For subsequent experiments we therefore chose the lowest BCP concentration that exerted a chondro-protective effect against CM, which was 1 µM. When chondrocyte cultures were treated with AA, from 2 to 125 µM both in the absence (Figure 1C) or in the presence (Figure 1D) of CM pretreatment, a significant decrease of cell viability was shown only with 125 µM without CM pretreatment. In contrast, 50 µM GlcN treatment significantly reduced cell viability with or without CM pretreatment (*p* < 0.05, Figure 1E,F). However, when GlcN (10 µM or 20 µM) was administered in the presence of 1 µM BCP and 2 or 10 or 50 µM AA (mixtures A1, A2 and A3) to chondrocyte cell cultures, the viability was comparable to that of control cell cultures (Figure 1G) and increased significantly in comparison to CM pretreated cell cultures for A1 and A2 mixtures (Figure 1H).

In accordance with these findings, most of the subsequent experiments were performed with the mixture of BCP, AA and GlcN at low doses (A1, 1 µM BCP, 2 µM AA and 10 µM GlcN; A2, 1 µM BCP, 10 µM AA and 20 µM GlcN; A3, 1 µM BCP, 50 µM AA and 20 µM GlcN).

### 2.2. BCP, AA and d-Glucosamine Mixture Induced a Reduction of ROS Formation in Human Chondrocytes Exposed to H_2_O_2_

**Figure 2:**
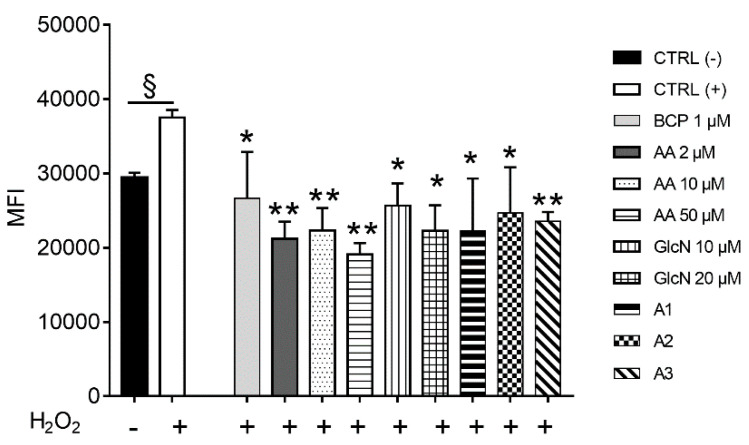
Reactive oxygen species (ROS) generated by human chondrocytes in the presence or absence of BCP, AA or GlcN, alone or in combination. Cells were cultured under optimal conditions, and intracellular ROS generation was evaluated using the H2DCFDA probe for 12 h and to 250 µM H_2_O_2_ for 2 h to induce intracellular ROS. Statistical differences are based on one-way ANOVA test with multiple comparisons. Data are reported as mean ± standard error (SE) of the percentage of fluorescence observed in least two independent experiments, each in triplicate. +, cell cultures exposed to H_2_O_2_; CTRL (-), untreated cell cultures; CTRL (+), cell cultures exposed to H_2_O_2_. * *p* < 0.05 and ** *p* < 0.01 compared with CTRL(+). MFI = mean fluorescence intensity.

Intracellular ROS generation was evaluated using the H2DCFDA probe following exposure to H_2_O_2_ for 2 h, in the presence or absence of all the molecules tested, alone or as mixtures A1, A2, or A3. As reported in Figure 2, the treatment of chondrocyte cultures with all the tested molecules revealed a significantly decreased level of ROS (*p* < 0.05) compared to the H_2_O_2_-treated human cells, which was higher (*p* < 0.01) for cells treated with AA. However, all the combinations of BCP, AA and GlcN (A1, A2, A3 mixtures) revealed a significant antioxidative effect in comparison to H_2_O_2_-treated chondrocytes, with A1 showing the lowest mean MFI and the A3 mixture exerting the greatest robust effect (*p* < 0.01).

### 2.3. BCP, AA and GlcN Mixtures Reduce Chondrocyte Expression of IL-1beta, NF-κB and MMP-13

**Figure 3:**
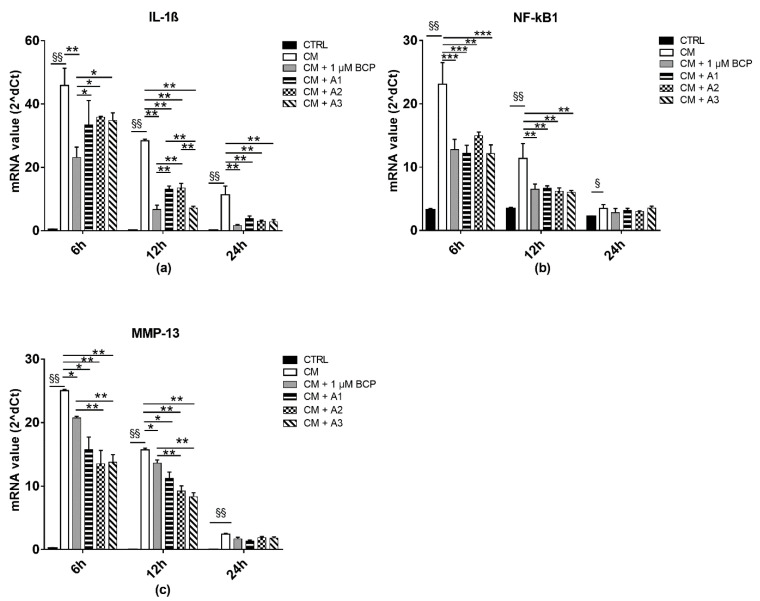
Chondrocyte expression of proinflammatory molecules and MMP-13 after exposure to U937 CM, in the presence or absence of BCP, AA and GlcN. Human chondrocytes were exposed for 24 h to CM of activated U937 and then cultured in the presence or absence of 1 μM BCP, alone or in combination with different amounts of AA and GlcN (A1, A2, A3 mixtures). RNA transcript levels specific for (**a**) IL-1β, (**b**) NF-κB-1 and (**c**) MMP-13 were evaluated by qPCR at 6, 12 and 24 h after treatment. Statistical differences are based on one-way ANOVA test, and data are expressed as mean ± SE obtained from three independent experiments. CTRL, untreated cells; CM, chondrocytes cultures exposed to U937 CM. * *p* < 0.05 and ** *p* < 0.01; *** *p* < 0.001 compared with CM treated control; § *p* < 0.05 and §§ *p* < 0.0001, CM-treated cells vs. untreated cells.

Activated U937 human monocytes are able to produce a wide variety of inflammatory molecules released in the culture medium, mimicking a natural inflammatory event. In particular, a significant upregulation of the proinflammatory cytokines *IL-1β, TNF-α, IL-6,* and *Gal-1* and *-3* was demonstrated (Figure S1). The anti-inflammatory effect of the BCP, A1, A2 and A3 mixtures was then tested on human chondrocyte cultures treated with the CM of activated U937 human monocytes for 24 h. The results showed that 1 µM BCP reduced the *IL-1β* expression from 6 h after its administration to CM-treated cells (Figure 3), and that its combination with AA and GlcN induced a significant downregulation not only of *IL-1β* gene expression, but also of *NF-*κ*B1* at 6 and 12 h after treatment (Figure 3a,b; *p* < 0.05, *p* < 0.01). Since proinflammatory cytokines and ROS may influence the expression of MMPs, the expression of *MMP-13* in human chondrocytes treated with the CM of activated macrophages was evaluated. Indeed, it was shown that the exposure of chondrocyte cultures to the CM induces an overexpression of MMP-13. However, 1 µM BCP, alone or in combination with all the tested combination with AA and GlcN (A1, A2, A3), significantly downregulated *MMP-13* (*p* < 0.05) (Figure 3c) in comparison to CM-treated control cell cultures from 6 to 12 h after treatment. In this case, a synergistic effect of BCP, AA and GlcN was highlighted at 6 and 12 h.

### 2.4. BCP, AA and GlcN Mixture Upregulate the Expression of Collagen Type II and Aggrecan

**Figure 4:**
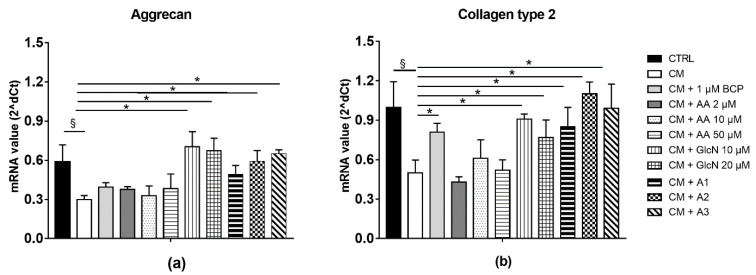
Aggrecan and collagen type II expression of chondrocyte cultures exposed to U937 CM in the presence or absence of BCP, AA and GlcN. Human chondrocytes exposed for 24 h to CM of activated U937 were cultured in the presence or absence of 1 μM BCP, alone or in combination with different amounts of AA and GlcN (A1, A2, A3 mixtures). RNA transcript levels specific for aggrecan (**a**) and collagen type II (**b**) were evaluated at the gene level by qPCR at 24 h after treatment. § *p* < 0.05 CM vs. the control and * *p* < 0.05 vs. CM-treated cells. Statistical differences are based on one-way ANOVA test, and data are expressed as mean ± SE obtained from three independent experiments. CM, chondrocytes cultures exposed to U937 CM.

Human chondrocyte cultures were treated with CM of activated U937 human monocytes, revealing a significant decrease of *collagen type II* and *aggrecan* gene expression (Figure 4). Gln treatment showed a preponderant effect in inducing the expression of *aggrecan* and *collagen type 2*. A2 mixture exhibited the highest effect in inducing *collagen type II* expression. However, a significant upregulation of the two cartilage ECM molecules was also found for cells exposed to all the tested mixtures (A1, A2, A3) in the 24 h CM-treated cells (Figure 4a,b, *p* < 0.05) but not to BCP alone, highlighting a consistent link between anti-inflammatory activity of the mixtures and an upregulation of *collagen type II* and *aggrecan* expression.

### 2.5. The Effect of BCP Is PPAR-Mediated

**Figure 5:**
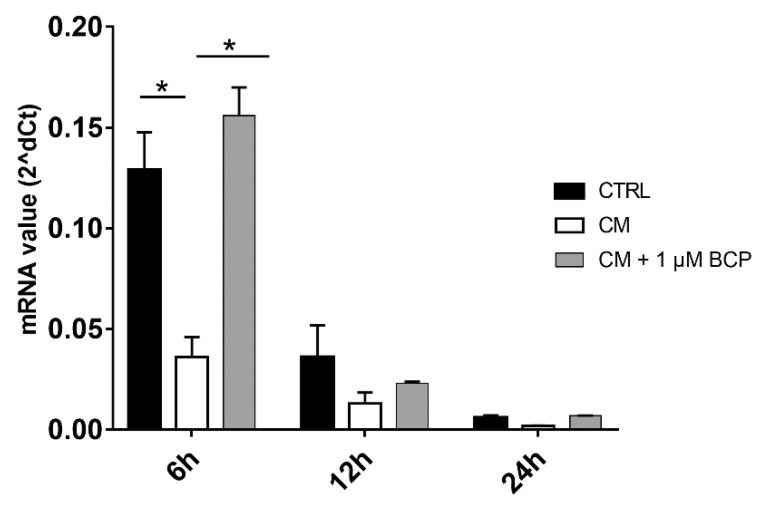
PPAR-γ expression of chondrocyte cultures exposed to U937 CM in the presence or absence of BCP. Human chondrocytes were exposed for 24 h to CM of activated U937 and then cultured in the presence or absence of 1 µM BCP. RNA transcript levels for *PPAR-γ* were evaluated by qPCR at 6, 12 and 24 h after treatment. Statistical differences are based on one-way ANOVA test, and data are expressed as mean ± SE obtained from three independent experiments. CTRL: untreated cells; CM: cell treated with CM of activated U937 cells. * *p* < 0.05.

To demonstrate that BCP acts through PPAR modulation, we analyzed the expression of this molecule in chondrocyte cultures after CM of activated U937 treatment. We found that PPAR-γ expression was significantly reduced by CM in comparison to cells incubated only with DMEM at 6 h after treatment (Figure 5). However, when BCP was added to cell cultures pretreated with CM, PPAR-γ expression was restored. At 24 h, the treatment with BCP restored PPAR-γ expression to normal levels.

## 3. Discussion

The present report demonstrated that the bicyclic sesquiterpene BCP exerts a protective effect on primary human articular chondrocyte cultures treated with the CM of activated U937 cells, and that this effect is more effective at low doses. There are several studies supporting that low doses of BCP can ameliorate cell viability [25,26], and others that report that this response against cytotoxicity is exerted not only at low but also at high concentrations in a biphasic mode [27].

The protective effect of the low concentration of BCP on CM-induced chondrocyte inflammation was enhanced when the molecule was administrated to cell cultures in combination with AA and GlcN at low doses. Moreover, it was found that BCP, alone or in combination with AA and GlcN, exerts protective effects against ROS and inflammation at lower concentrations than those reported in previous studies with lower possible toxicities. Importantly, all three of the compounds are considered safe dietary supplements. BCP has been approved as a food additive by the United States Food and Drug Administration (USFDA) and the European Food Safety Authority (EFSA) [28,29]. AA is considered a safe compound, and many authors have stated that the use of GlcN helps maintain normal joint cartilage [10,30,31]. However, recent investigations have reported an increase in adverse reactions related to high doses of food supplements and drug treatments [32,33]. For this reason, the reduction of the doses of molecules administrated as pharmacological therapies would offer a valuable perspective for exploring new potential therapeutic strategies in the treatment of OA that deserve to be further investigated. A recent study reported that the action of BCP on neural cell viability is CB2-receptor-mediated, and the addition of a CB2 receptor antagonist to cell cultures completely abolishes the effect of BCP [34]. Since it is well demonstrated that human chondrocytes express CB2 receptor [35], it is possible that the effect of BCP on chondrocyte viability might be mediated by cannabinoid receptors, though further studies are needed to explore the exact mechanism of its action.

This study demonstrated, for the first time, to the best of our knowledge, that BCP exerts in vitro an anti-inflammatory effect on inflamed chondrocyte cultures at low doses. Indeed, 1 µM BCP alone could decrease the *IL-1β* levels. Importantly, BCP in combination with low doses of AA (from 2 to 50 µM) and GlcN (10 or 20 µM) led to the downregulation of *NF-*κ*B*, *IL-1β* and *MMP-13* gene expression. NF-κB1 is activated by exposure of cells to LPS or inflammatory cytokines [3], and recent studies have shown that it is active in several cell types [36], playing unexpected roles in the regulation of gene expression. Other studies demonstrated that NF-κB plays a key role in inducing, in turn, cytokine expression and cytokine-induced secretion of MMPs, since its inhibitors could significantly block the induction of MMP-1 and -13 [37,38]. The findings reported here demonstrated that BCP, AA and GlcN decrease the expression of *IL-1β* and *NF-*κB; thus, it can be hypothesized that the anti-inflammatory effect of this mixture of molecules is mediated by NF-κB1. Importantly, the mixtures A1, A2 and A3 had a greater effect on *NF-*κB1 and *MMP-13* expression at 6 and 12 h compared to the BCP, supporting the use of the mixtures compared to the single molecule alone.

Regarding ECM molecules, the A1, A2 and A3 mixtures determined an increase of *aggrecan* and *collagen type II* expression at 24 h. An increase of the expression of these molecules was also obtained by GlcN alone, but to a minor extent. AA acts as a scavenger molecule for ROS, reducing the oxidative stress damage, and also as an anti-inflammatory molecule. The exact mechanisms by which GlcN exert its protective effects are not fully understood, whereas the mechanism of action of the BCP has only been partly clarified. In fact, BCP is an agonist of CB2 receptor and directly activates PPAR-α [39,40] or, indirectly, through the CB2 receptor, the PPAR-γ pathways [41]. Thus, it may be speculated that some of the biological functions of cannabinoids, such as anti-inflammatory effects, are mediated by the activation of PPAR. Indeed, a recent study provided evidence that BCP significantly ameliorates the severity of arthritis in mice, reducing the expression of pro-inflammatory cytokines through a cross-talk between CB2 and PPAR-γ receptors [14,42]. It was recently reported that AA not only acts as a scavenger for ROS, but it also has anti-inflammatory and chondro-protective effects, stimulating collagen type II and aggrecan synthesis [43]. However, a different line of evidence has emerged from in vivo studies about the effects of GlcN in inhibiting the progression of OA [10,21]. This study confirmed the ability of GlcN to inhibit the expression of cytokines and, indirectly, demonstrated its antiarthritic properties as an anti-inflammatory agent in combination with BCP and AA.

## 4. Materials and Methods

### 4.1. Drugs and Chemicals

BCP was purchased from Biosfered (Turin, Italy), ascorbic acid (AA) from Sigma (St. Louis, MO, USA) and GlcN from Alfa Aeser (Kandal, Germany). A total of 500 mM BCP was diluted with absolute ethanol. AA and GlcN were dissolved in distilled water at, 280 mM and 10 mM, respectively. The solutions were prepared immediately before administration to cell cultures.

### 4.2. Primary Human Chondrocyte Culture

Articular chondrocytes were isolated from joint cartilage biopsies of OA patients who underwent total knee or hip replacement at the Orthopedic and Traumatologic Clinic (University-Hospital of Padova) following a standard procedure [44]. Cartilage biopsies were collected from each patient after receiving informed consent and in accordance with the Local Ethical Committee of Padova Hospital (AOP1617). Briefly, cartilage was chopped finely then treated first with 0.25% trypsin for 15 min (Invitrogen, Carlsbad, CA, USA) and finally overnight at 37 °C with type I collagenase (100 U/mL, Worthington Biochemical). The digested material was then resuspended in Dulbecco’s modified Eagle’s medium (DMEM) containing 10% fetal bovine serum (FBS), 1% penicillin/streptomycin (P/S), 1% glutamine (all from GIBCO) and 50 µg/mL ascorbic acid [44].

The released cells were counted manually and expanded until passage 3. The complete medium was renewed daily and the viability of the cells was tested by Trypan Blue staining. The expression of CB2 receptor by chondrocytes was demonstrated by quantitative PCR (qPCR) analysis (data not shown).

### 4.3. Activated U937 Monocyte Conditioned Medium

Human monocytes U937 (Thermo Scientific, Wilmington, DE, USA) were differentiated to macrophages by the addition of phorbol 12-myristate 13-acetate (PMA) (Sigma-Aldrich) at a final concentration of 50 ng/mL for 48 h then 1 µg/mL lipopolysaccharides (LPS, Sigma, St. Louis, MO, USA) in complete RPMI medium (containing 10% FBS, 1% P/S, 1% glutamine, all from GIBCO) for 1 h. Cells were then washed and cultivated with complete RPMI for 24 h to produce the inflammatory conditioned medium (CM), which was collected, filtered using a filter with a pore diameter of 0.22 µm (Millipore, MA, USA) and used to treat human chondrocyte cultures [12]. The differentiation of monocytes to macrophages was verified under an inverted phase-contrast microscope and confirmed with mRNA expression of the macrophage differentiation marker CD68 (Figure S2).

### 4.4. Evaluation of the Effects of BCP, AA and GlcN on Human Chondrocyte Viability

The effects of BCP, in the presence or absence of AA and GlcN mixtures, were assessed on chondrocyte viability. Cell cultures were treated with increasing quantities of the compounds, alone (BCP, from 0.5 to 50 µM; AA from 2 to 125 µM; GlcN from 10 to 50 µM) or in three different combinations (A1, 1 µM BCP, 2 µM AA and 10 µM GlcN; A2, 1 µM BCP, 10 µM AA and 20 µM GlcN; A3, 1 µM BCP, 50 µM AA and 20 µM GlcN). In all experiments, chondrocytes were seeded at a density of 7000/cm^2^ in multiwell dishes. Viability was determined at 1, 3 and 6 days by the MTT test (3–4,5-dimethylthiazol-2-yl-2,5-diphenyltetrazolium bromide, Sigma, MO, USA) using a modified Denizot method [45]. Briefly, cells were seeded in multiwell dishes and incubated with fresh medium or CM for additional 24 h. The cells were treated with the tested compounds and cultivated until assessment of viability after 1, 3 and 6 days, removing the culture medium before adding the MTT reagent. With this procedure, only viable cells with functioning mitochondria can oxidize MTT to a violet-red reaction product.

The BCP, AA and GlcN concentrations for cell culture treatments were chosen after a preliminary in vitro viability test. Subsequently the effect of selected concentrations of BCP, AA and of GlcN were tested on human chondrocyte cell cultures. The effect of the small amounts of ethanol in the BCP solution on cell viability (from 0.001 to 10%) was tested on chondrocyte cultures before their treatment with the tested molecules (Figure S3).

Data are reported as mean of three independent experiments using cell cultures obtained from three different donors, each performed in triplicate.

### 4.5. Analysis of Antioxidative, Anti-Inflammatory and Regenerative Effects Induced by BCP, AA and GlcN

The effects of BCP, AA and GlcN at different concentrations on human chondrocytes exposed to U937 CM for 24 h were assessed at different times by the detection of reactive oxygen species (ROS) generation analysis and by the analysis of proinflammatory molecules expression.

#### 4.5.1. Detection of Intracellular ROS Generation

Human chondrocyte cultures were treated with BCP, AA and GlcN or their mixture for 12 h; prior to the end of the treatment, cells were exposed to 250 µM H_2_O_2_ for 2 h to induce intracellular ROS production. Cell cultures were trypsin-harvested and the generation of intracellular ROS was measured using 2′,7′-dichlorodihydrofluorescein diacetate (H2DCFDA; molecular probes: Invitrogen, Carlsbad, CA, USA). H2DCFDA is a nonfluorescent probe that is rapidly oxidized to the fluorescent 2′,7′-dichlorofluorescein in the presence of intracellular ROS. Briefly, after washing the cells from trypsin and medium, cells were loaded for 30 min at 37 °C with 5 µM H2DCFDA in warm PBS, washed twice and placed in 60 µL of PBS. Fluorescence was measured in a BD FACSCanto™ flow cytometer (Becton, Dickinson and Company, Franklin Lakes, NJ, USA) at excitation and emission wavelengths of 485 and 535 nm, respectively [12]. Each experiment was performed in triplicate.

#### 4.5.2. RNA Isolation and qPCR Analysis

Total RNA was extracted by TRIzol (Life Technologies, Carlsbad, CA, USA) according to the manufacturer’s instructions. The RNA quality was controlled using a Nanodrop 2000c spectrophotometer (Thermo Scientific) measuring absorbance at 260/280 nm. A total of 500 ng of total RNA was reversely transcribed using oligo-dT and Superscript II (Life Technologies, Carlsbad, CA, USA), according to the manufacturer’s instructions. The qPCR was performed on a Rotor-Gene RG-3000A (QIAGEN, Germany) using Xpert fast SYBR (GRISP, Portugal) [12]. The expression of *NF-*κ*B*, *IL-1β*, *IL-6*, *TNF-α*, *Gal-1*, *Gal-3*, *MMP-13*, *collagen type II*, *aggrecan* and *PPAR-γ* was analyzed by qPCR. Primers used for qPCR analysis are listed in Table 1. The qPCR data were compared to the housekeeping gene Peptidylprolyl isomerase A (*PPIA*), to normalize the results. Gene expression was then evaluated with 2^d*C*t method, where d*C*t = *C*t peptidylprolylisomerase A (PPIA) − *C*t target gene.

### 4.6. Statistical Analyses

Statistical analyses were performed using the unpaired Student’s t-test and the one-way analysis of variance (ANOVA) test with Dunnett’s multiple comparison post hoc test, using GraphPad Prisma 7 (San Diego, CA, USA). A *p* < 0.05 was considered as statistically significant.

## 5. Conclusions

In conclusion, BCP alone exerts an anti-inflammatory effect and decreases the oxidative stress, AA decreases the ROS production and GlcN increases the production of extracellular matrix components. The combined use of the three substances determines a synergistic effect on inflamed chondrocytes. In particular, there is a decrease of oxidative stress, of key anti-inflammatory cytokines (such as IL-1β) and of the levels of MMPs. Importantly, there is an increase of extracellular matrix component production (such as aggrecan and Col II). All these effects suggest that the combined use of the BCP, AA and GlcN could be a promising and interesting treatment to counteract cartilage degradation in OA.

## Figures and Tables

**Table 1:** Genes investigated and primers used.

Gene (Accession Number)	Name	Primer Sequences
ACAN (NM_001135.3)	Aggrecan	Fw 5′-TGCGGGTCAACAGTGCCTATC-3′ Rv 5′-CACGATGCCTTTCACCACGAC-3′
Col2A1 (NM_001844.5)	Collagen Type II Alpha 1 Chain	Fw 5′-CGCTGGTGCTGCTGACGCTGCTCGT-3′ Rv 5′-GGCACCTTTTTCACCTTTGTCAC-3′
IL-6 (JQ250825.1)	Interleukin 6	Fw 5′-CACGCCTTGGACAGAATCCA-3′ Rv 5′-CCTCCAGCAACCAGGAATGT-3′
IL-1β (NM_000576.3)	Interleukin 1 beta	Fw 5′-GAATCTCCGACCACCACTACAG-3′ Rv 5′-TGATCGTACAGGTGCATCGTG-3′
LSGALS1 (NM_002305.4)	Galectin 1	Fw 5′-TCTCGGGTGGAGTCTTCTGA-3′ Rv 5′-GTTCAGCACGAAGCTCTTAGC-3′
LSGALS3 (NM_002306.4)	Galectin 3	Fw 5′-CTGCTGGGGCACTGATTGT-3′ Rv 5′-TGTTTGCATTGGGCTTCACC-3′
MMP-13 (AY741163.1)	Matrix metalloproteinase 13	Fw 5′-AACGCCAGACAAATGTGACC-3′ Rv 5′-AGGTCATGAGAAGGGTGCTC-3′
NF-κB-1 (NM_001243985.2)	nuclear factor kappa B subunit 1	Fw 5′-CCGGGATGGCTTCTATGAGG-3′ Rv 5′- GGGGTTGTTGTTGGTCTGGA-3′
PPAR γ (NM_001330615.2)	peroxisome proliferator activated receptor gamma	Fw 5′-ACCCAGAAAGCGATTCCTTCA-3′ Rv 5′-AGTGGTCTTCCATTACGGAGAGATC-3′
PPIA (NM_021130.5)	Peptidylprolyl Isomerase A	Fw 5′-GGGCTTTAGGCTGTAGGTCAA-3′ Rv 5′-AACCAAAGCTAGGGAGAGGC-3′
TNF-α (NM_000594.3)	TNF- alpha	Fw 5′-AAGCCTGTAGCCCATGTTGT-3′ Rv 5′-GGACCTGGGAGTAGATGAGGT-3′

Fw = forward; Rv = reverse.

## Data Availability

The data presented in this study are available on request from the corresponding author.

## Supplementary Materials

The following are available online at https://www.mdpi.com/1424-8247/14/3/286/s1, Figure S1: The conditioned medium (CM) of activated U937 cells induced pro-inflammatory molecules, Figure S2: CD68 expression of activated U937 human monocytes, Figure S3: The effect of ethanol concentration on human chondrocyte viability.
