# Supplementary material for: In Vitro Effects of Low Doses of β-Caryophyllene, Ascorbic Acid and d-Glucosamine on Human Chondrocyte Viability and Inflammation
Source: Pharmaceuticals (Basel). 2021 Mar 23;14(3):286. doi: 10.3390/ph14030286 (PMC8005039; doi:10.3390/ph14030286)
Supplement: Supplementary file 1 [file pharmaceuticals-14-00286-s001.pdf]

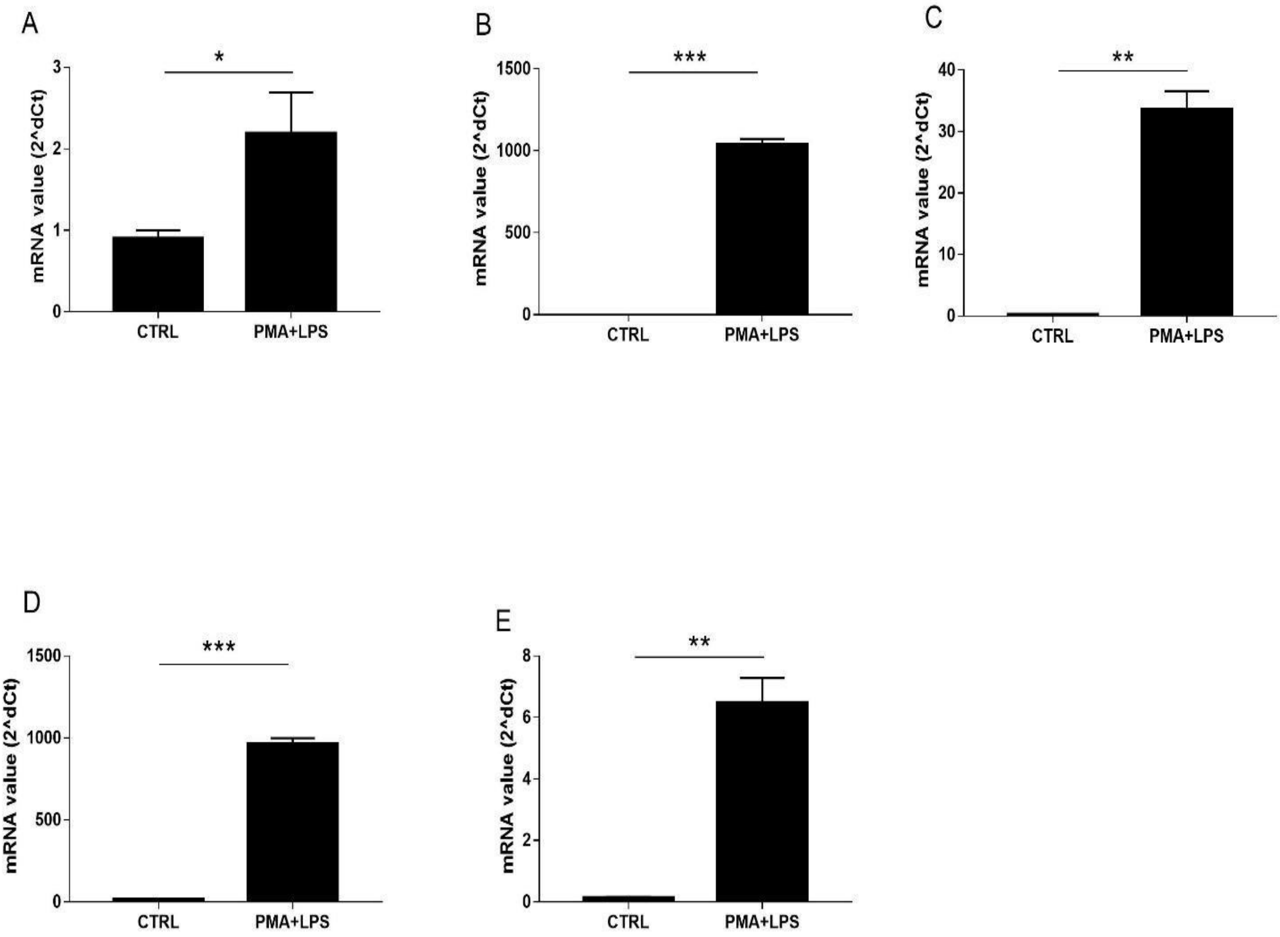

S1. The conditioned medium (CM) of activated U937 cells induced pro-inflammatory molecules. Detection of pro-inflammatory molecules in U937 cells exposed for 24h to PMA and LPS and then cultured under optimal conditions. RNA transcript levels specific for A), *IL-6*, B) *IL-1β*, C) *TNF-α*, D) *Gal-1* and E) *Gal-3* were evaluated by qPCR. Data are expressed as mean ± SE obtained from three independent experiments. Statistical differences based on unpaired Student's t-test. *P<0.05, **P<0.01 and ***P<0.001 vs untreated cells.

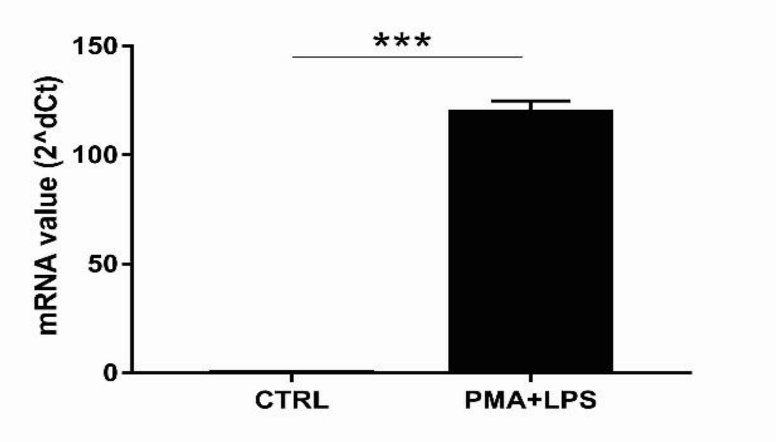

S2. CD68 expression of activated U937 human monocytes. Cells were exposed for 24 h to CM of activated U937 cells and then the mRNA expression was evaluated by qPCR analysis. Data are expressed as mean ± standard error (SE) of 3 independent experiments. Statistical differences based on unpaired Student's t-test. ***P<0.001 vs untreated cells.

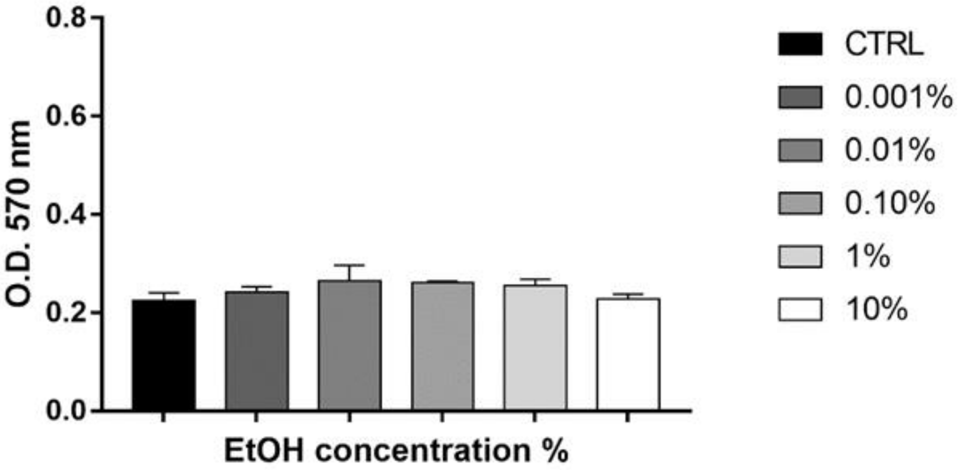

S3. The effect of ethanol concentration on human chondrocyte viability. Cells were seeded in 96-well culture dishes and exposed to ethanol (EtOH). The MTT test was performed 48 h after treatment. Data are reported as mean ± SE of three independent experiments. Statistical differences are based on ANOVA test with multiple comparison.
